# Love is expensive: the impact of initiating versus terminating romantic relationships on the onset of obsessive-compulsive disorder

**DOI:** 10.1017/S1092852925000033

**Published:** 2025-01-31

**Authors:** Donatella Marazziti, Leonardo F. Fontenelle, Gustavo A. de-Medeiros, Maria Eduarda Moreira-de-Oliveira, Gabriela B. de Menezes, Manuel Glauco Carbone, Stefania Palermo, Lara Foresi Crowther, Francesco Weiss, Riccardo Gurrieri, Alessandro Arone

**Affiliations:** 1 Department of Clinical and Experimental Medicine, Section of Psychiatry, University of Pisa, Pisa, Italy; 2 Saint Camillus International University of Health and Medical Sciences, Rome, Italy; 3 Obsessive, Compulsive, and Anxiety Spectrum Research Program, Institute of Psychiatry of the Federal University of Rio de Janeiro (IPUB/UFRJ) & D’Or Institute for Research and Education (IDOR), Rio de Janeiro, Brazil; 4 Department of Medicine and Surgery, Division of Psychiatry, University of Insubria, Varese, Italy

**Keywords:** Obsessive-compulsive disorder, Romantic love, Romantic relationships, Y-BOCS

## Abstract

**Introduction:**

Recently, some observational studies suggested that romantic love (RL) might influence the phenotypic expression of obsessive-compulsive disorder (OCD). The aim of the present study was to investigate the impact of different stages of RL on the clinical expression of OCD.

**Materials and Methods:**

Two hundred and twelve patients with OCD onset related to the development or the termination of a romantic relationship (RR) and who were attending outpatient units at the University Psychiatric Clinic of Pisa, Italy, and seven specialized OCD clinics in Brazil were recruited. The assessment instruments were: the Structured Clinical Interview for DSM-5 Disorders (SCID-5), the Yale OCD Natural History Questionnaire, and the Yale-Brown Obsessive-Compulsive Scale (Y-BOCS). Participants were divided into two groups (love-precipitated [LP-OCD] and break-up OCD [BU-OCD]).

**Results:**

The total Y-BOCS and obsessions and compulsions subscales scores were similar and indicative of severe OCD in the two groups. The average age of onset was significantly lower in the BU-OCD group, perhaps reflecting a vulnerability of the brain’s maturational stages to “undesirable” events in young individuals at risk for OCD. A trend towards aggression and symmetry, and ordering and rearrangement dimensions in BU-OCD patients emerged, possibly reflecting an amplification of some normal features of a RR.

**Conclusions:**

Our findings suggest that different stages of RL may influence some features of OCD, namely the age of onset and specific dimensions. Again, RL poses the risk of developing this pathological condition in vulnerable individuals. Further research on the topic should be encouraged.

## Introduction

The constant interest in obsessive-compulsive disorder (OCD) of the last decades, due to its high prevalence^1^ and treatment resistance^2^, has promoted increasing studies to elucidate its specificities. Although the gathered data have contributed to a greater understanding of its pathophysiology, the factors, and events favoring the onset of OCD, which is quite heterogeneous, are still unclear.^3^ Several individuals report OCD onset shortly after a stressful event, however, this concept results are too broad, given the extensive range of possible triggers, and the individual vulnerability depending on genetics, character, temperament, lifestyle, environment, and resilience.^4^ ^,^^5^

More recently, a focus was directed toward the impact of romantic relationships (RR) on OCD, whether as a precipitating (“pathogenic”) or a modulating (“pathoplastic”) factor possibly influencing its phenotypic expression.^6^ ^,^^7^ However, it is a matter of debate whether love-precipitated OCD (LP-OCD) represents or not a distinct clinical variant of this disorder. One of the first studies dates back to the end of the last century, when a certain overlap between some characteristics of early romantic love (RL, that is the early phase of falling in love) and the symptoms of OCD was highlighted, in particular, the persistence of thoughts and intrusive images, heightened awareness of a loved one, selective shifting of the focus and the obsession towards trivial features of an experience.^8^ ^,^^9^ Another shared feature involves exaggerated senses of responsibility and fear for the safety of the loved one, similar to the increased sense of commitment and responsibility commonly found in pair bonding.^10^ Indeed, the fear of harm is a typical dimension of the OCD subtype related to aggression.^11^ ^,^^12^ In a similar fashion, fantasies, and desires on sexual themes are typical of falling in love^13^, which follows the wave of the dimension of sexual and religious obsessions of OCD. Such statements do not imply that these features of falling in love are pathological themselves, but rather that they recall similar symptomatic dimensions of OCD.

Sternberg’s triangular theory of love (1986) emphasizes the role of the sense of incompleteness at the basis of the drive that leads to a RR. Specifically, this sense of incompleteness, with shades of imperfection and insufficiency, may contain features of longing for wholeness with roots in either RL or, in some cases, in parental love.

Some authors offered an evolutionary explanation, as RL would represent a behavior preserved throughout human history, aimed at the formation of pair bonding^10^, while considering that the choice of a stable partner requires characteristics of specificity and reciprocity. Such an important aim cannot be achieved by serendipity, but rather it requires specific neurobiological systems continuously subjected to influence and adjustments during the individual’s life.^14^ ^–^^16^

Indeed, the feeling “love” in a RR undergoes physiological “ups and downs,” given that the obsessive thoughts typical of the first phase are exhausting and consuming resources that could be employed in other more evolutionarily advantageous behaviors. Alterations in these mechanisms have been postulated to represent the bridge between obsessive thoughts in RL and the pathogenesis of OCD^10^ that therefore would share common neurobiological underpinnings belonging to the so-called social brain.^17^ ^,^^18^ Interestingly, a reduced density of the platelet serotonin (5-HT) transporter (SERT) was noted in both individuals at the early phase of RL love, subjects with OCD, as compared with healthy and not-in-love controls that returned to normal levels after 12 and 18 months.^8^

The scientific literature also includes anecdotal and case reports supporting these findings, as that of a law student who had traced the onset of OCD, characterized by obsessions of symmetry and contamination, immediately after every time he fell in love, with remission following the break-up.^6^

More recently, a study investigated the relationship between RL and OCD in a large sample of 981 participants. The results of this research demonstrated an important association between LP-OCD and two clinical variables: the late onset of obsessions and the severity of sensory phenomena, such as feelings of incompleteness.^16^

Given the paucity of available information, the present study was conceived to investigate the impact of RL on socio-demographic features and clinical expression of OCD. The main objective was to explore the possible characteristics of OCD depending on whether its onset had occurred at the beginning or following a RR break-up. We will use the expressions LP-OCD and break-up OCD (BU-OCD) to denote the OCD onset, respectively, at the beginning and after the end of a RR. We hypothesized that, due to the different features of the trigger event in the two groups, the rates of comorbidity, especially in the spectrum of mood and anxiety disorders, might differ, with a prevalence of the former in BU-OCD and the latter in LP-OCD.

## Methods

### Sample recruitment

The study included 212 patients of both sexes (121 men and 91 women, mean age 35.24 ± 12.35 years) attending outpatient units at the University Psychiatric Clinic of Pisa, Italy, between 2022 and 2023 and seven specialized OCD clinics across different Brazilian universities between 2003 and 2009, all included in follow-up databases. The inclusion criteria for this specific study were being between 18 and 65 years of age, fulfilling the criteria for a diagnosis of OCD according to the Structured Clinical Interview for DSM-5 Disorders (SCID-5)^19^, and with the onset of the disorder coinciding or occurring shortly (within 2/3 months) after falling in love or at the end of the RR. Age under 18 years or over 65 years, patients unable to sign an informed consent, and patients who reported the onset of OCD after triggers or events not correlated with romantic relationships or seemingly no factor, were exclusion criteria. After a complete description of the study, a written informed consent was obtained from each subject to participate in the study that was approved by the Ethics committee (USP # 968/05; UNIFESP # 302/2006; UFRGS # 06/171; IPA # 6600023; and UFRJ # 0024.0.249.000-06).

### Assessment scales

#### Structured clinical interview for DSM-5 disorders (SCID-5)

The Italian translation of the SCID-5-CV was used in the Italian sample, whereas a Portuguese translation of its older counterpart, the SCID-IV, was employed in the Brazilian subjects. Of note, because the diagnostic criteria for the assessed disorders (including major depressive disorder [MDD], dysthymic disorder, bipolar disorders type I and II [BDI and BDII], OCD, generalized anxiety disorder [GAD], social anxiety disorder [SAD], and panic disorder) were essentially the same, we felt they could be comparable. However, as the diagnostic criteria of PTSD changed substantially from DSM-IV to DSM-5, a decision was made not to report and compare its prevalence between the two groups.

#### Yale OCD natural history questionnaire

The Yale OCD Natural History Questionnaire is a short-term self-report questionnaire used to investigate events and factors that may influence the clinical picture of OCD.^20^ ^,^^21^ In this research, this tool was used to evaluate whether the RR was a precipitating factor for OCD, specifically whether the participants had experienced obsessive-compulsive symptoms for the first time when they were in love or, instead, following a breakup. This questionnaire was also used to investigate the age of onset of OCD and its developmental course. A Portuguese version and an Italian version were used according to the country of origin.

#### Yale-brown obsessive-compulsive scale (Y-BOCS)

The Y-BOCS is a clinician-rated semi-structured interview with two main sections: a symptom checklist (made of 67 items) and a 10-item severity scale further divided into subscales that separately assess the severity of obsessions (5 items) and compulsions (5 items). Each item is rated on a 5-point Likert-type scale, with scores from 0 (indicating no symptoms) to 4 (extreme severity of the symptoms), by covering different domains (time spent on symptoms, distress, interference from symptoms, and resistance and control over symptoms). The Y-BOCS scores for obsessions and compulsions range from 0 to 20, whilst the Y-BOCS total score is included in a 0–40 range.^22^ ^,^^23^ AS Brazilian patients were assessed with the Dimensional version of the Y-BOCS (D-YBOCS)^11^, an effort was made to establish the presence of OCD symptoms using the original Y-BOCS as a reference. Specific language-tailored versions of the Y-BOCS were used according to the country of origin.

### Statistical analysis

The whole sample was analyzed both as a whole and when divided into two groups, LP-OCD and BU-OCD, according to the romantic event triggering OCD.

All demographic and clinical data were presented for continuous variables in terms of mean ± SD and variation range (min and max values) when required. Categorical variables were expressed as frequencies (numbers) and percentages. The Kolmogorov–Smirnov test was used to determine the normality of the distribution of the variables. Comparisons for continuous variables were performed with the independent-sample Student’s *t*-test for variables that follow a normal distribution. Comparisons for categorical variables were conducted using χ^2^ test (or Fisher’s exact test when appropriate).

The correlations were explored by calculating the Pearson’s correlation coefficient or Spearman rank correlation. Pearson’s correlation was used to measure the degree of the relationship between linearly related variables. For Pearson’s correlation, both variables had to be normally distributed (normally distributed variables have a bell-shaped curve). Other assumptions included linearity and homoscedasticity. Linearity assumes a straight-line relationship between each of the two variables, and homoscedasticity assumes that data is equally distributed about the regression line.

Spearman’s rank correlation is a nonparametric test that is used to measure the degree of association between two variables. The Spearman’s rank correlation test does not carry any assumptions about the distribution of the data and is the appropriate correlation analysis when the variables are measured on a scale that is at least ordinal. The assumptions of the Spearman’s correlation are that data must be at least ordinal and the scores on one variable must be monotonically related to the other variable. In this study, *p*-values lower than 0.05 were considered statistically significant.

The whole sample was analyzed both as a whole and when divided into two groups, LP-OCD and BU-OCD, according to the romantic event triggering OCD.

## Results

### Total sample

Thirty-eight (17.9%) patients were from Italy and 174 (82.1%) were from Brazil. One hundred-twenty were single (56.6%), 70 were married (33%), 13 were divorced (6.1%), five had a stable relationship (2.4%), and four were widowed (1.9%). Considering the employment status, 105 participants had a job at the time of the evaluation (49.5%), while 107 were unemployed (50.5%). The clinical diagnosis showed a high prevalence of comorbid psychiatric disorders. Specifically, 79 individuals were affected by major depressive disorder (MDD) (37.3%), 22 by bipolar disorder (BD: 7 by BD-I, 3.3% and 15 by BD-II, 7.1%), and 19 by dysthymic disorder (10%). The most frequent anxiety disorders were generalized anxiety disorder (GAD: n. 68, 32.1%) and panic disorder (26, 12.3%). Substance abuse disorder (SUD) was recorded in 47 patients (22.2%).

The mean age of onset of OCD in the global sample was 15.61 ± 7.30. One hundred sixty-eight (80%) and 44 (20%) patients had, respectively, an insidious and an acute onset. One hundred seventy-three (81.6%) subjects had a chronic course and the remaining 39 (18.4%) were episodic. The mean Y-BOCS total score of the sample was 25.48 ± 8.11, the mean score for the obsession subscale was 12.81 ± 4.06, and that of the compulsion subscale was 12.67 ± 4.51. The most common symptom dimensions were, in decreasing order: miscellaneous, symmetry/ordering/rearrangement, contamination/washing, aggression, sexual/religious, and hoarding (Table 1).

**Table 1.** Y-BOCS Total, Obsessions and Compulsions Scores (mean + SD) (panel a) and OCD Symptoms (panel b) in the Total Sample

Panel a		
Total score	25.48	8.11
Obsessions score	12.81	4.06
Compulsions score	12.67	4.51

Panel b			
Dimension	Symptoms	n	%
Aggression	Present	137	64.6
	Absent	75	35.4
Sexual/ religious	Present	121	57.1
	Absent	91	42.9
Contamination/ washing	Present	146	68.9
	Absent	66	31.1
Symmetry, ordering, nd rearrangement	Present	160	75.5
	Absent	52	24.5
Hoarding	Present	95	44.8
	Absent	117	55.2
Miscellaneous	Present	186	87.7
	Absent	26	12.3

### *Subgroups (LP-OCD and BU-OCD*)

We then divided the whole sample in the two groups, LP-OCD and BU-OCD, according to the romantic event triggering OCD. The LP-OCD group consisted of 152 (66 men and 86 women) individuals (71.7%) and the BU-OCD of 60 (25 men and 35 women) (28.3%). One hundred and twenty-three (80.9%) and 29 subjects (19.1%) were, respectively, from Brazil and Italy within the LP-OCD group, and 51 (85%) and nine (15%), respectively, from Brazil and Italy in the BU-OCD group.

### Socio-demographic features of LP-OCD and BU-OCD

The mean age of the LP-OCD group was significantly higher than that of the BU-OCD group (37.01 ± 12.95 vs. 31.47 ± 10.01; *t* = 3.302, *p* = 0.001). As shown in Table 2, the comparison of the socio-demographic data did not reveal any significant intergroup differences other than current age (Table 2).

**Table 2.** Demographic Features of the Two Subgroups

	LP-OCD		BU-OCD	
	n = 152		n = 60	
	n	%	n	%
*Sex*				
Male	66	43.4	25	41.7
Female	86	56.6	35	58.3
*Country*				
Italy	29	19.1	9	15.0
Brazil	123	80.9	51	85.0
*Marital status*				
Single	91	59.7	29	48.3
Married	47	31.0	23	38.3
Stable partner	3	2.0	2	3.3
Widowed	3	2.0	1	1.7
Divorced	8	5.3	5	8.4
*Employment stat*us				
Employed	79	52.0	26	43.3
Unemployed	73	48.0	34	56.7

### Comorbidity features of LP-OCD and BU-OCD

In the LP-OCD group, 56 individuals were affected by MDD (36.7%), 51 by GAD (33.5%), 30 by SAD (19.7%), 16 by panic disorder (10.5%), 15 by dysthymic disorder (9.9%), 10 by BDII (6.6%), and 5 by BDI (3.3%). In the BU-OCD group, 23 patients received a diagnosis of MDD (38.3%), 17 of GAD (28.3%), 17 of SAD (28.3%), 10 of panic disorder (16.7%), 4 of BDII (6.7%), 3 of dysthymic disorder (5%), and 2 of BDI (3.3%). No statistically significant intergroup differences were detected.

### Clinical features of LP-OCD and BU-OCD

The mean age of onset of OCD was statistically higher in the LP-OCD than in the BU-OCD group (16.57 ± 7.364 vs. 12.95 ± 6.82, t = 3.188, p = 002). One hundred and seventeen (77%) subjects of the LP-OCD had an insidious OCD onset and 35 (23%) of the BU-OCD group showed an acute one, a51 (85%) subjects of the BU-OCD group had an insidious onset, and nine (15%) an acute onset. As for the course of disease, 126 subjects (82.9%) had a chronic course and 26 (17.1%) an episodic one in the LP-OCD group. In the BU-OCD group, 47 (78.3%) participants showed a chronic course of the disease and 13 (21.7%) an episodic one.

### Obsessive-compulsive symptoms of LP-OCD and BU-OCD

In the LP-OCD group, the mean Y-BOCS total score was 25.41 ± 8.687, that of the obsessions scale was 12.74 ± 4.365, and that of the compulsions scale was 12.67 ± 4.812. In the BU-OCD group, the mean Y-BOCS total score was 25.86 ± 6.621, that of the obsession scale 13.07 ± 3.248, and that of the compulsion scale was 12.80 ± 3.764 (Table 3), with no intergroup differences. The correlations between OCD symptoms dimensions and the two groups showed just a trend towards higher aggression (χ^2^ = 2.784, *p* = 0.095) and symmetry (χ^2^ = 2.813, *p* = 0.093) in the BU-OCD group (Table 4).

**Table 3.** Socio-Demographic Data and Y-BOCS Total Scores of LP-OCD and BU-OCD

	LP-OCD		BU-OCD	
	n = 152		n = 60	
*Y-BOCS scores*	Mean	SD	Mean	SD
Total score	25.41	8.687	25.86	6.621
Obsessions score	12.74	4.365	13.07	3.248
Compulsions score	25.86	6.621	12.80	3.764

**Table 4.** OCD Symptoms Dimensions in LP-OCD and BU-OCD Individuals

	LP-OCD		BU-OCD	
	n = 152		n = 60	
	n	%	n	%
Aggression	92	60.5	45	75.0
Sexual/religious	84	55.3	37	61.7
Contamination/washing	106	69.7	40	66.7
Symmetry, ordering and rearrangement	111	73.0	49	81.0
Hoarding	67	44.1	28	46.7
Miscellaneous	55	86.0	55	91.0

## Discussion

It has been suggested that LR may act as a vulnerability factor or a trigger of different psychopathological disorders, including OCD.^8^ ^,^^24^ ^–^^32^ Therefore, the primary aim of this study was to explore the possible differences in the clinical pictures of OCD based on its onset with the beginning of a RR (LP-OCD) or following its conclusion (BU-OCD).

When we analyzed the characteristics of the two groups, some interesting findings emerged. The socio-demographic characteristics were comparable, with a marked prevalence of participants from Brazil and a slightly greater representation of the female gender in both groups. However, our original hypothesis that the comorbidity patterns could be different in the two groups, with the beginning of a RR being accompanied more by more relevant anxious symptoms, thus possibly resulting in full-blown anxiety disorders, due to its features of attraction and falling in love, was not confirmed.

Similarly, we were unable to confirm the hypothesis that patients of the BU-OCD group with the onset of the disorder secondary to the stressful and emotional relationship break-up, would have shown more severe depressive/irritable symptoms developing into mood disorders. Nonetheless, our results highlighted that, albeit the most frequent comorbidities in the two groups were MDD, GAD, and SAD, no significant intergroup differences emerged. In a recent study in patients reporting the onset of OCD in the “in-love” phase, as compared with individuals with OCD precipitated by other events, a slight prevalence, albeit not significant of mood and anxiety disorders was found in the former group.^16^ ^,^^33^ These similar comorbidity patterns might be simply due to the fact that mood and anxiety disorders are the most frequent comorbid conditions in OCD, rather than being associated with the experience of love *per se* ^16^ ^,^^34^ Previous studies had also highlighted a significant association between RL and anxious-depressive symptoms in clinical samples of healthy subjects and not amongst treatment-seeking patients with OCD.^35^ ^,^^36^ This finding might suggest that healthy individuals and those with an underlying psychopathological vulnerability might differently experience such symptoms.

Again, the age of OCD onset was significantly lower in the BU-OCD than in the LP-OCD, which may perhaps be the mirror of a vulnerability of the brain’s maturational stages to “undesirable” events in young individuals at risk for OCD. Similarly, a trend towards two types of obsessions and compulsions (aggression and symmetry, ordering and rearrangement) in the BU-OCD group emerged, possibly reflecting an amplification of some normal features of a RR.

To our knowledge, this is the first study evaluating the different impact between the start of RL and break-up on OCD, and there is no published data to compare our findings. Regardless, we hypothesize that the increased susceptibility to the effects of a breakup may be the mirror of an increased vulnerability of the brain’s maturational stages to “undesirable” life events in young individuals who are at risk for OCD. Indeed, due to cerebral immaturity, the brain is basically more sensitive to different types of insults, thus reflecting only a partial formation of the underlying neural substrates.^37^ This is particularly true for the prefrontal cortex (PFC), some areas belonging to the limbic system and the white-matter-associated fibers that undergo a progressive and heterogeneous maturation from early puberty up to the age of 20.^38^ In particular, the development of the PFC includes the dorsal-lateral prefrontal circuit, which is responsible for executive and goal-oriented behaviors and for reward evaluation, the orbito-frontal PFC, involved in behavioral processes, and the medial prefrontal circuit involved in motivation.^38^ The limbic system matures at the same time as the PFC with parallel changes in the monoaminergic systems.^38^

The limbic system also exerts a significant influence on sexual processes and behaviors, which also mature over time. All these areas are supposed to play a role in both OCD and RL.^39^ ^,^^40^ Moreover, responses to stress vary during adolescence, thus contributing to its wide heterogeneity of mental disorders.^41^ ^,^^42^ Therefore, an early RR disappointment or break-up, may turn out in a “tragedy” on neural developing substrate, and favor the onset of OCD in susceptible individuals.^24^ ^,^^31^ ^,^^32^ This would also be in line with the evidence that relationship break-ups represent a frequent cause of concern during adolescence, as they are associated with emotional distress and worsening of mental well-being, possibly leading to depressive symptoms, self-harm, or even suicide.^43^ ^,^^44^

We were unable to identify any other differences between LP-OCD and BU-OCD. Indeed, there seem to be some controversies in the literature. The study of McLauchlan and colleagues a few years ago identified a greater frequency of episodic onset in LP-OCD^16^, at variance with other findings.^7^ As McLauchlan et al.’s (2022) and our results suggest, this discrepancy might originate from the methods of selecting participants in the study. The first sample consisted of subjects who reported an onset of OCD when falling in love, while Thompson et al. recruited heterogeneous individuals who were in love, who had started an intimate relationship and were about to get married.^7^

The Y-BOCS total and obsessions/compulsions subscale scores were similar in the LP-OCD and BU-OCD, while indicating no influence of the RR on the overall severity. On the contrary, previous data reported an association between LP-OCD and symptoms of sensory phenomena^16^, that are mental and physical sensations that may occur either before or with repetitive behaviors, such as feeling of incompleteness, which are commonly reported by the patients as a driver to compulsions and were also found to be predictors of greater severity of OCD symptoms.^45^ ^,^^46^

The analysis of the correlations demonstrated a trend close to significance between obsessions and compulsions, namely aggression, and symmetry/ordering/rearrangement in the BU-OCD group. From a neuroevolutionary perspective, the trend towards greater rates of fear of harm following a relationship break-up may mirror the persistence of affiliative bonds through the development of heightened sense of responsibility. We, therefore, hypothesize that the physiological circle of thoughts and worries, and often actions, oriented to warrant the partner’s safety during a RL, may persist beyond the original scope, while resulting in a full-blown fear of harming others when the relationship ends. This concept would be further strengthened in cases of onset of OCD after a break-up in early adolescence by virtue of the immaturity of the circuits responsible for the regulation of goal-oriented behaviors and reward evaluations.^38^ In any case, tailored studies would be desirable to confirm or not this hypothesis. In a similar fashion, the trend towards symmetry/ordering symptoms in the BU-OCD group might share a neuroevolutionary ground. This OCD dimension is characterized by the need for things to look good and balanced, extreme anxiety levels in case of asymmetry, meticulous ensuring of symmetry, accurate arranging of personal belongings/items/clothes, often intertwined with perfectionism and events of “magical thinking”.^47^ During a RL, it is common that one of both partners try to ensure that “everything is under place” for the relationship to last, possibly with need of control and perfectionism and with the aim of maintaining the control over the territory shared by the two. After a break-up, despite the disappearance of the loved one, the concerns and behaviors oriented towards this “just right” dimension persist, although directed towards other aspects of the subject’s life, thus perpetuating such a structured and methodical shape of thinking and behaviors.

Finally, we were unable to identify a relationship between sexual/religious symptoms with BU or LP OCD. Arguably, sexual obsessions and compulsions are highly heterogeneous, including unwanted thoughts or images about others, but also doubts about one’s own sexuality, even in the absence of real reasons. Importantly, sexual symptoms were not examined in isolation, but together with symptoms with religious content, which could add to the heterogeneity and explain negative findings. It could be argued that this subtype of OCD would be more likely to arise in the search for motivations and explanations after a relationship break-up, especially in the younger age groups subject to a hormonal storm that, on an immature brain, could lead to a full-blown psychopathology. However, our data indicates that sexual/religious symptoms seem as likely to occur in LP-OCD and, for this reason, be related to RL in general regardless of earlier versus later in OCD.

The interpretation of our findings requires some caution. First of all, our sample included subjects with an OCD diagnosis who were actively seeking help for this disorder. As such, it is possible that there may be an overrepresentation of more severe forms of this disorder (as shown by the mean high Y-BOCS) scores, and, as such, our findings cannot be perhaps generalized to less severe forms of OCD. We have also to mention the role of cultural heterogeneity as a potential limitation, although the influence of Italian ancestry and culture in Brazil should not be minimized. Moreover, the distinction in two subgroups was based on the Yale OCD Natural History Questionnaire, which retrospectively evaluates the triggers and events behind the onset of OCD. Therefore, although a recall bias about previous onsets cannot be excluded, much attention was given to the circumstances surrounding the onset of OCD. As such, extending the age inclusion criterion of our sample to younger individuals <18 years of age, might lead to further intriguing findings. Moreover, the cross-sectional design of our study, whilst useful to shed light on the association between the independent and dependent variables considered, does not allow us to draw particular inferences on causal links. On a similar line, reverse causality could not be excluded.

Future studies should also include an evaluation of family history and, particularly, the presence of cases of OCD and RL-precipitated OCD within the relatives. Similarly, the comparison of the treatments used, whether psychotherapy or pharmacological or both, may be useful. Nevertheless, the overall severity of OCD seems to be related to either falling in love or a break-up. Again, the investigation of RL may be strengthened by the use of more tailored assessment instruments, such as the Passionate Love Scale, which specifically explores the cognitive, emotional, and behavioral components of passionate love.^48^ Larger samples are also desirable to increase the size of our findings.

## Conclusions

Our research was the first to systematically investigate the influence of different stages of RR (falling in love vs. breaking up) on the clinical picture of OCD. The present findings, although not confirming our main hypothesis, led to intriguing data. Our results showed an earlier age of onset of OCD following the end of a romantic relationship as compared with its beginning, thus indicating the influence of RR on the onset of OCD.

We also found a trend towards symptoms with aggressive and symmetry content in the group experiencing OCD after a RR break-up. Our explanation is that, given the earlier age of onset in these patients, a stressful and potentially traumatic event such as a romantic break-up in early adolescence may favor the persistence of thoughts and behaviors oriented at increasing the length of the RR, due to the immature of the neurobiological substrates underlying motivation, reward and goal-oriented behaviors in this age group.

Our results overall suggest that different stages of RR may influence some features of OCD, namely the age of onset and specific dimensions, with no impact on the overall severity of the clinical picture. These findings, together with current literature, should encourage further research to better clarify the impact of RR on OCD. They also define some of the features of these individuals and how love, one of the most natural experiences of humankind, may pave the way for the onset and specific features of OCD in vulnerable individuals especially with a certain degree of brain immaturity. This is not surprising, as love induces neurochemical changes that are frankly, albeit transient, “pathological.”
